# From Vulnerability to Stability: Migrant Nurses' Experiences of Autonomy, Competence and Relatedness—A Qualitative Descriptive Study

**DOI:** 10.1155/jonm/8260066

**Published:** 2025-03-02

**Authors:** Princess Villamin, Violeta Lopez, Deependra Kaji Thapa, Michelle Cleary

**Affiliations:** ^1^School of Nursing, Midwifery & Social Sciences, CQUniversity, Sydney, New South Wales, Australia; ^2^Department of Epidemiology and Biostatistics, School of Public Health, Indiana University, Bloomington, Indiana, USA

**Keywords:** internationally qualified nurse, migrant nurse, migration, qualitative descriptive study, regional migration, regional nursing workforce, retention, self-determination theory

## Abstract

**Aim:** To understand how migrant nurses perceive their needs for autonomy, competence and relatedness are satisfied and relate how these contribute to regional workplace retention.

**Design:** A qualitative descriptive study.

**Methods:** Data were collected through semistructured interviews among 17 migrant nurses employed at a hospital in regional Australia. Interviews were transcribed verbatim and analysed using reflexive thematic analysis. The self-determination theory provided a framework for the study.

**Results:** One overarching theme, facing challenges with determination to make oneself at home, was identified, with themes: migration and relocation to a regional area, commencing and adjusting to the workplace and integrating with the community. These are further explained with subthemes: experiencing personal vulnerabilities, experiencing familial challenges and adjustment, building connections, finding one's feet, finding meaningful work through nurse empowerment, valuing relationships at work, and embracing the regional lifestyle.

**Conclusion:** An unfamiliar work environment and culture may challenge migrant nurses' needs for autonomy, competence and relatedness, potentially impacting their integration. Supportive work environments, valued workplace relationships, community support and networking with peers from similar backgrounds can assist migrant nurses successfully transition, which may impact long-term retention.

**Summary**

• **Implications for the profession:** The results of this study highlight migrant nurses' perceptions of how their experiences satisfy their needs for autonomy, competence and relatedness.
• This knowledge may aid organisations employing migrant nurses in identifying and implementing strategies that may mitigate migrant nurses' transition challenges, which may contribute to workforce and community integration and long-term retention.
• **Reporting method:** This study adhered to Standards for Reporting Qualitative Research guidelines [1].

## 1. Introduction

The global healthcare sector faces considerable challenges from the chronic nursing shortage [2]. Poor retention rates due to burnout, exacerbated by an ageing workforce and ongoing nurse migration, further challenge workforce stability [2–4]. The increased migration is contributing to nurses transferring more readily between host and source countries, resulting in difficulties retaining nurses, including in regional and rural areas [5–7]. This adds to the complexity of nurse migration and retention, with some countries utilising migration schemes to recruit nurses to regional areas [8–10]. The ongoing mobility of nurses has also increased workforce diversity, where at least 4%–49% of a country's workforce comprises migrant nurses [11], making it more challenging to identify and implement retention strategies as migrant and nonmigrant nurses may require different retention strategies [10].

A scoping review on migrant nurse retention and turnover reported the necessity of considering individual needs and motivations in the context of nurses' source and host countries, aside from the classic push and pull factors, to understand what retains nurses postmigration [12]. One motivation theory that may provide this perspective is the self-determination theory (SDT) [13]. This theory identifies three basic psychological needs (BPNs): autonomy, competence and relatedness, which are fundamental to facilitating high-quality motivation, which, in turn, may contribute to positive workplace outcomes, including job satisfaction and decreased turnover intention [14, 15]. Understanding how migrant nurses perceive their needs for autonomy, competence and relatedness may provide vital information to inform organisations in modifying environmental and contextual factors. These modifications may contribute to fostering high-quality motivation among migrant nurses, increasing retention and decreasing turnover. This research describes migrant nurses' transition experiences to understand how they perceive their needs for autonomy, competence and relatedness are satisfied and how these experiences contribute to regional workplace retention.

## 2. Background

SDT is a motivational theory that posits that individuals experience optimal motivation and well-being when their environment supports the satisfaction of their needs for autonomy, competence and relatedness [13, 16]. Satisfaction of the needs for autonomy (sense of volition or self-endorsement of one's actions), competence (sense of mastery and effectiveness) and relatedness (sense of belonging and connectedness) lead to overall development, optimal functioning and high-quality motivation, while frustration can lead to ineffectiveness, ill-being and adverse outcomes [17, 18]. Among nurses, the satisfaction of BPNs may contribute to career commitment and satisfaction [19] and work engagement [20, 21] and may decrease turnover intention [21, 22]. Satisfaction of BPNs among nurses has also been found to mediate the relationship between personal strength and depressive symptoms [23], work factors and job satisfaction [24] and organisational support and work engagement [20].

SDT has established the universality of BPNs such that the positive outcomes from satisfying the needs for autonomy, competence and relatedness remain constant across cultures [25, 26]. Thus, despite the possibility that studies on migrant nurses may include nurses from various cultural backgrounds, the BPNs remain relevant. The link between satisfaction of BPNs and positive organisational outcomes and the universality of BPNs supports the relevance of exploring migrant nurses' experiences of autonomy, competence and relatedness and how they perceive these experiences contribute to workplace retention in a regional area.

Although there is research on nurses and their experiences of autonomy, competence and relatedness, these are explored in different contexts without the consideration of migration status [27–29]. Migration may cause individuals to experience stressful situations as they adjust to their new environment. Migrant and nonmigrant nurses may also have different perceptions of how their BPNs are met. For example, nurses may perceive satisfaction of their need for competence when they engage in meaningful tasks, have self-efficiency and receive positive feedback [29]. While migrant nurses may have the same perceptions, satisfaction of their need for competence may be compounded by challenges to their professional competence related to differences in practice, unfamiliar cultural norms and language barriers. They may have the technical skills to practice proficiently; however, practising and adjusting to a new working environment in a different country may be challenging [30]. They may also experience doubts from their colleagues in terms of their clinical proficiency and have limited professional opportunities, which may challenge the satisfaction of their need for competence [31, 32]. Migrant nurses may have different and possibly more challenging workplace experiences than nonmigrant nurses; thus, exploring their experiences may provide valuable insights into which factors foster or thwart the satisfaction of their BPNs and how these contribute to regional workplace retention.

## 3. The Study

### 3.1. Aim

This qualitative study aimed to understand motivations and identify factors affecting the overall motivation of migrant nurses, leading to workplace retention in a regional area. This aim has resulted in two foci of the study: migrant nurses' motivations to migrate and relocate to a regional area (the first focus) and migrant nurses' experiences contributing to their retention in a regional area (the second focus). As the study resulted in findings that are not practical to convey in a single article and due to the differences in the focus of the findings, the researchers have reported the findings separately. This paper focuses on migrant nurses' experiences, how they perceived that their needs for autonomy, competence and relatedness were satisfied, and how they perceived that their experiences contributed to regional workplace retention (the second focus). A companion paper describing migrant nurses' intrinsic and extrinsic motivations to migrate and relocate to a regional area is reported elsewhere [33].

### 3.2. Design

A qualitative descriptive design was employed to enable researchers to obtain first-hand perceptions of participants' experiences of autonomy, competence and relatedness and how they perceived that these experiences contributed to their intentions to stay in a regional area. This design allowed researchers to gain a straightforward albeit deep understanding of events while staying close to participants' experiences [34].

The researchers adopted a constructivist paradigm and approached this study with the assumption that there are multiple realities (relativist ontology) and that these realities are co-constructed between the participants and the researchers, which are ultimately shaped by individual experiences (subjectivist epistemology) [35, 36]. This aligns with the underlying philosophy of qualitative description [37–39].

The SDT [13] was adopted as the guiding framework for the study. This motivation theory was chosen due to robust evidence that the satisfaction of BPNs contributes to positive organisational outcomes regardless of the participants' cultures. The SDT was used to frame part of this study's research question and inform the interview guide and discussion. To avoid making the findings ‘fit' the SDT, the analysis was conducted via a data-driven rather than a concept-driven approach.

### 3.3. Study Setting and Recruitment

Participants were recruited using purposive and snowball sampling from one regional hospital in Australia. Participant recruitment was facilitated by support from the hospital's Director of Nursing and delegate. The hospital's Director of Nursing sent an initial contact email to introduce the researcher, the project and relevant details to all nurses at the study site. This was followed by an advert containing the same content posted on official communication boards in each nursing department and throughout the hospital. Although recruitment was facilitated by the hospital's Director of Nursing and their delegate, the interviewer (researcher) was not employed at the study site. This mitigated any power imbalance.

Purposive sampling ensured participants met the following inclusion criteria: (1) current employment as a nurse at the study site, (2) completion of initial nursing qualification in any country apart from Australia, and (3) migration to regional Australia within the last 7 years. Participants born overseas but completed their initial nursing qualification in Australia were excluded. Snowball sampling [40] through referrals of initial participants enabled the researcher to access participants who met the inclusion criteria. The researchers used snowball sampling rather than respondent-driven sampling (RDS) as there was no need to track social ties and referrals, as in the case of RDS [41].

### 3.4. Data Collection

One interviewer (PV) conducted individual, semistructured interviews between November 2023 and February 2024. The research team developed the interview guide, informed by the literature and SDT [13], to elicit participants' perceptions of their experiences of autonomy, competence and relatedness. Despite organisational approval to attend the interview during work hours, only two elected to participate within their work hours. Interviews were conducted via telephone or videoconferencing. The time and medium of the interview were of the participants' choosing, adding to their autonomy during their participation [42].

Interview questions included experiences of migrating to Australia as a nurse, differences and similarities between participants' current and previous clinical/nursing experiences, their experiences during transitions into the community and workplace, their relationships within and outside of the workplace and their (including their family as applicable) overall migration experience. The interview guide was reviewed by the team, pilot-tested (by PV), with the pilot interview reviewed (by MC) before data collection. These pilot interviews were excluded from the final count, analysis and reporting. No questions were modified as a result of the pilot interview. See Appendix S1 for the interview guide for this study.

This paper reports on interview responses to several primary questions from the semistructured interview guide: What steps did you take to migrate to Australia as a nurse? What challenges did you anticipate before moving to Australia, and how did you prepare for these? What challenges did you face, and how did you overcome them? What strategies or support outside of employment helped you overcome these challenges? Which clinical area are you currently working in? What support did you receive to help you adjust in your workplace? What has been your experience working as a nurse in Australia? Describe your thoughts and feelings surrounding your desire to continue to stay in your workplace. Describe how your workplace relationships and interactions affect you. In general, how would you describe your experience as a migrant in Australia? What is your spouse's (and child's/children's) experience after moving to Australia? Is there anything else you would like to say to help me understand how your experiences influence your intention to stay in your current workplace and place of residence? Probes and prompts as per the interview guide were used when applicable to elicit further data and clarify meaning. A companion paper [33] reports participants' responses to other questions in the interview guide which are primarily about their motivations to migrate.

The interviews were between 45 and 60 min, were audio recorded and transcribed verbatim. The transcripts were proofread before commencing the analysis. Data collection and analysis were simultaneous, and further data collection ceased when the participants' responses were replicated. Data saturation was achieved after the 14th interview, during which the entire transcript could be coded into codes already developed during the earlier stages of analysis and coding rounds. However, to prevent prematurely ceasing data collection, further three interviews were conducted, although no new concepts or codes were developed from the subsequent interviews. At this point, the team was satisfied that more interviews would not add further value or lead to new concepts. Data saturation is critical in achieving rigour in qualitative research, ensuring researchers do not report findings from ‘thin' data [43].

### 3.5. Data Analysis

The transcripts were analysed using reflexive thematic analysis [44, 45] in an inductive (data-driven) approach. Although thematic and content analysis both offer the level of interpretation required in qualitative descriptive studies [39, 46, 47], this study used thematic analysis as it did not aim to quantify the findings, rather present nuanced patterns across the data [47]. Qualitative content analysis is also often considered atheoretical or aligning with (post)positivist assumptions [48], which is not congruent with the aim of this study. Identifying codes and themes from the participants' responses provided answers to the research questions and enabled an accurate representation of the responses. In line with Braun and Clarke's [44, 48] six-step approach to reflexive thematic analysis, the analysis for this research included (1) data familiarisation, (2) initial coding, (3) generating initial themes, (4) developing and reviewing themes, (5) refining, defining and naming themes and (6) writing the report.

Transcripts were proofread against the original recordings to check for accuracy, after which initial thoughts were annotated while re-reading transcripts to enable familiarisation and gain a sense of the data [49]. NVivo 14 [50] enabled a systematic coding process where similar data fragments were grouped until the entire transcript was coded. Researchers (PV, MC, VL) then reviewed the codes and discussed ways to cluster them to generate potential themes. These themes were checked repeatedly to ensure that they represented the coded extracts. Conflicting, contradictory or redundant themes were collapsed or separated into another theme. Themes were reviewed to identify the overarching theme, the main themes and the subthemes. After repeated rounds of reviewing themes, subthemes and codes within themes, theme names were defined to capture the ‘essence' of each theme [44]. Themes and subthemes were given descriptive names to give readers an immediate sense of their central concepts. The analysis process was nonlinear and recursive. The final themes resulted from repeated engagement with the transcripts, ensuring the themes were coherent and answered the research question.

### 3.6. Ethical Considerations

This study was approved by the WA Country Health Service Human Research Ethics Committee (project reference number RGS0000006301) and the Central Queensland University Human Research Ethics Committee (approval number 0000024004). All participants were provided with a participant information sheet detailing what their participation entails, including voluntary participation and withdrawal from the research, provided it was within 7 days of their interview. All participants provided informed written consent, with research data stored in accordance with the University's ethics requirements. Participants were addressed using their selected pseudonyms throughout the interview, and findings were reported by participant numbers.

### 3.7. Rigour and Reflexivity

Lincoln and Guba's [51] criteria of credibility, dependability, confirmability and transferability provided the framework for rigour. Using probes during participant interviews, regular research team meetings and debriefings, and the inclusion of participant quotations in the findings enhanced the credibility, while providing sufficient details of the study setting and context enhanced transferability. Member checks were not performed as participants may be unable to recognise their interview responses from the synthesis of all interview data while also avoiding to impose on participants' schedules [52, 53]. As an alternative to member checks, the researcher (interviewer) probed during interviews and repeated participant responses to clarify meaning and avoid misunderstanding or assumptions [39, 52]. Dependability and confirmability were ensured through an audit trail, which provided a rationale for the decisions made throughout the study. Interviews were conducted by one researcher which maintained consistency and contributed to the study's dependability. The researchers acknowledged the potential for bias throughout the study and mitigated this using reflexivity and memoing in addition to regular team supervision.

## 4. Findings

### 4.1. Characteristics of the Sample

Seventeen participants were interviewed for the study. The ages of participants were between 30 and 58 years, with their clinical experience ranging between 7 and 37 years. Participants were mostly female (*n* = 11), married (*n* = 14), with their children living with them (*n* = 12). On average, the participants' length of stay in the regional area was 3 years, ranging between 1 and 7 years. A few participants migrated directly from their source country (*n* = 4), while some migrated from host countries (*n* = 5), and from another Australian state after relocating from their source country (*n* = 8). The host countries that participants migrated from were Ireland (*n* = 1), the Middle East (*n* = 3) and the United Kingdom (*n* = 1). Participants' source countries included India (*n* = 6), Kenya (*n* = 1), New Zealand (*n* = 1), the Philippines (*n* = 7), Scotland (*n* = 1) and South Africa (*n* = 1).

### 4.2. Themes

**Figure 1:**
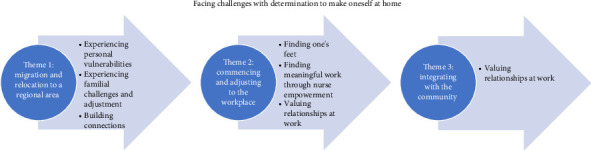
Migrant nurses' migration journey: themes and subthemes.

Participants' experiences were grouped into an overarching theme: *Facing challenges with determination to make oneself at home* (in the host country). This overarching theme is grouped into main themes to represent migrant nurses' experiences during three transition periods in their journey leading up to their intention to stay in the regional area: (1) migration and relocation to a regional area, (2) commencing and adjusting to the workplace and (3) integrating with the community. These themes are further supported by seven subthemes: (1) *Experiencing personal vulnerabilities,* (2) *Experiencing familial challenges and adjustment,* (3) *Building connections,* (4) *Finding one's feet,* (5) *Finding meaningful work through nurse empowerment,* (6) *Valuing relationships at work* and (7) *Embracing the regional lifestyle* (Figure 1).

#### 4.2.1. Theme 1: Migration and Relocation to a Regional Area

##### 4.2.1.1. Experiencing Personal Vulnerabilities

Most participants reported feeling vulnerable after migrating, with their most common source of vulnerability stemming from financial challenges. Some described having to borrow large sums of money to migrate to Australia, so settling their debts while navigating their new environment was reported as a considerable hurdle. P15 described, ‘*It took a lot of money before I could get my registration as a nurse*' and ‘*the biggest concern [now] was how am I going to pay this off*.' P7 recalled, ‘*the agency [that lent money] is a loan shark requiring you to pay [a] high interest rate*'. P8 expressed a similar experience, having to ‘*work overtime to the point [of] working 18 hours a day just to make ends meet*'. Financial problems were also a challenge, even for participants who did not have debts to settle. P1 recalled migration being a ‘*big financial cost*' where ‘*in the early days, we just had no money to feed our kids or anything*'. The ‘*financial implication*' (P10) of moving resulted in participants working ‘*double shifts, [including working] night shift [to] a late shift*' to ‘*get some income*' (P1) ‘*because we need to get as much work*' so ‘*we chose whichever [work was] available in the hospital*' (P14). P7 added skipping holiday celebrations, stating, ‘*I'd rather be on shift and get double pay*'. P5 stated, ‘*I always just think I need the money because we've got bills to pay*'.

Participants also had to learn to be ‘*independent*' (P3) and ‘*do it on* [their] *own*' (P6) as they steered away from a culture that ‘*was a little bit close[r] together*' because ‘*the people around have their own life*' (P6). This cultural and lifestyle adjustment resulted in feelings of ‘*loneliness*' (P7) even among participants who migrated with families as they still had to leave their extended families and established social networks behind. P4 stated, ‘*We [were] lonely [because] we didn't have anyone*', with many participants seeking to establish new connections and ‘*work really hard outside of work to make friends*' (P1). This, however, was not without its challenges as recounted by P1, ‘*forming new relationships when you've come from a different background can be quite hard*'. P2 noted that ‘*it's hard when your whole lifestyle has changed*'. P17 described ‘*[feeling] a bit homesick from time to time*', but added, ‘*that's going to happen wherever you are, wherever you move to*'.

##### 4.2.1.2. Experiencing Familial Challenges and Adjustment

Some participants had to migrate alone and leave their families behind while ‘setting up' in Australia. P8 described having to ‘move first to get established', and P16 echoed ‘*coming [for] the first time with the whole family [would be] very hard*'. P10 described the initial migration period as a ‘*difficult time*', recalling, ‘*[my family] call[ed] and sa[id] Oh please come back home. Why leav[e] us?*”, adding ‘*it was not an easy time*'. P14 recalled, ‘*my daughter was one year old when I left [her, along with] my husband*', leaving P14 to navigate living in Australia ‘*alone*'.

As participants navigated their challenges, they also supported their families, who were facing challenges of their own. Challenges were apparent for spouses whose qualifications were not accepted, which limited their professional opportunities. Participants' spouses worked across engineering, healthcare, agriculture, industry, commerce and information technology. However, upon relocation, they could not practice in line with their profession as their qualifications ‘*were not accepted by Australia*', making them feel ‘*uneducated in a way*' (P1). P9 added that despite ‘*[having] a Master's and [doing] a globally accepted course, [my husband] didn't really get a [similar] job*'. Participants' spouses ‘*found it really hard to get work*' (P2), leaving them feeling ‘*frustrated*' (P14) at having to work other jobs such as a ‘*cook*' or ‘*taxi [driving]*' as they often ‘*[don't] do well when not working*' (P2). Other participants' spouses had to ‘*change professions*' (P16) and work as a ‘*post[man]*', ‘*patient staff assistant*', ‘*patient care assistant*', ‘*coordinator*' and ‘*sterilisation technician*'.

Participants understand that similar to their process of acquiring nursing registration, their spouses may have to convert their qualifications to Australian qualifications. However, this was reported as a tedious process and, for some, often not feasible, especially in the early stages of transition. P16 stated, ‘*[my husband] need[s] to go [to] a remote site do a supervisory practice, but I will be alone [with] the kids, and I am doing full-time work*'. P12 added, ‘*[my husband] was stuck at home, like as childcare because I was working 5 days a week*'. Participants who had spouses who were nurses in their home countries often remained within the healthcare profession, working as ‘*patient care assistants*', ‘*carers*', ‘*disability support workers*', and ‘*sterilisation technicians*' instead of as nurses.

Aside from the inability to practice in alignment with their profession, some participants' spouses had to adjust without their social networks. P12 recalled, ‘*[my husband] was sad [to lose] a lot of friends*' while P15 stated, ‘*[my wife] was crying day and night, missing everybody back home*', adding ‘*it was lots of sadness moving here*'. Some participants' spouses anticipated the need for a new community connection, with P11 stating that ‘*my husband managed to get lots of friends before [we came] by joining [a] cricket group [online]*', aiding with their adjustment.

However, most participants reported that their children often ‘*didn't face any troubles*' (P13) adjusting and had no difficulty transitioning. P17 stated, ‘*Kids are so much more resilient*' because they ‘*are able to tolerate [transition] easily*' (P10). All participants with children mentioned that their children were happy, especially in terms of their education, with participants stating ‘*it's much easier here*' (P8) because ‘*they don't have much homework*' (P4) compared to their source countries where there is ‘*too much pressure [to] study*' (P16). However, P1 recalled an adjustment period where, ‘*I remember our children standing in front of the mirror trying to speak like an Australian, [and] they did that for years*' as they tried to ‘*assimilate*' into the community.

##### 4.2.1.3. Building Connections

All but two participants migrated to the regional area with little knowledge of what to expect, where to go or how to begin their lives. Most participants sought help and connections through social networks. These relationships, primarily with people from the same source country as theirs, were described to be crucial in helping them transition into the community. P16 recalled, ‘*I contacted one of my friends [who was] also here, so [I was] added to our Indian community group. Through that community, I [said] I needed temporary accommodation, then one of the families responded, [so] I stayed with them for 1 month*'. P11 added, ‘*One of our friends is in the city, so we got a contact number of [another] Indian friend [who] helped [us] when we [came]*'. P5 recalled struggling for ‘*the first 3 months, but after building connections and [meeting] a lot of Filipinos, it became easy*'. P15 stated, ‘*It's like one Filipino helping another Filipino*' when describing connections gained from one person to another that helped throughout the transition period. P10 remembered connecting with an old friend who became ‘*like a point man, [giving] me very important information*' that was of assistance.

For participants who did not have anyone to connect with, employer assistance, mainly through nurse unit managers (NUM), was reportedly vital in getting them acquainted with available support. Managers were the primary ‘go-to person' who helped them by linking them to people from the same source country and/or providing free accommodation until they found their place to live. P8's manager was ‘*trying to [arrange] a link with the Filipino community*' while P11 was introduced to their ‘*[clinical nurse specialist] from staff development, [who] was Indian*', adding ‘*my NUM assisted me with all the things*'. P14 recalled being told by the manager that someone ‘*[who] is going to start [in the same hospital] as well knows [me]*', so the ‘*hospital [placed] us in the same house*'. P12 remembered the difficulty in getting a house, ‘*so [the hospital gave] accommodation of 6 weeks, [for] free*', stating, ‘*the relocation that I got was a great support*'. Similarly, P13 was provided with ‘*a house, for free*' while receiving support from an ‘*[Indian] community who provide[d] us with everything*', so it did not become ‘*much [of] a struggle to move*' to the regional area.

All participants gradually formed workplace relationships that they identified as helping them adjust. P3 stated, ‘*When I first moved here, I did not know that it's essential to have a car, so [my colleagues] would give me lifts from home to work, and [they would] cook me food too*'. P3 added, ‘*They know that I don't have stuff to use, so they were helping me buy household stuff*'. P1 recalled thinking, ‘*There's much better connections in a smaller place, and small places will embrace you way more than a big place*' when discussing an easier transition into a regional area than a metropolitan area. P15 was taught ‘*the ins and outs, the do's and don'ts from a Filipino couple [who] were both nurses at the hospital*' while ‘*trying to get things together*'. P17 met an ‘*amazing family [who] really helped us overcome the challenge [of adjusting]*', feeling ‘*lucky to meet a few families*' that helped them throughout the transition.

#### 4.2.2. Theme 2: Commencing and Adjusting to the Workplace

##### 4.2.2.1. Finding One's Feet at Work

All participants, regardless of the years of their clinical experience, had a period of adjustment to their current clinical areas. The pathway by which participants gained Australian registration was identified as critical in how they adjusted. Some participants who gained reciprocal registration without undertaking bridging or conversion courses described feeling underprepared. P4 stated, ‘*When you do the bridging programs, you know something about Australia, but when you come directly from one country to a new country, you don't know the rules and regulations of these countries*'. P12 hoped ‘*whoever [employs] staff from overseas provide an orientation to the system for those who [are] coming directly without doing an adaptation program*'. ‘*Language barrier*' (P5) was also a challenge because ‘*even though we all spoke English, the words we used didn't have the same meaning in Australia as [they] did for us*' (P1). P4 thought colleagues assumed that ‘*we have migrated with that experience, but they don't understand that everything, even the chart, escalation, forms, was new for me*', so minimal support was given, adding ‘*I [felt] I was failing so [I wanted] to go back to the UK*'.

Other participants remained ‘*extra conscious*' (P10) despite having undergone bridging or conversion programs because of differences in practice, including their new-found ‘*autonomy*' (P7) and ‘*free[dom] to speak*' (P16). P7 recalled being able to ‘*initiate bloods and medications*', essentially ‘*building autonomy rather than wait for the doctor's order*'. While this practice is ‘*empower[ing]*' (P7), P6 reinforced the ‘*need to be cautious*' because ‘*here you're doing everything on your own*', whereas previously, ‘*we double-check everything, even oral medications*'. P3 felt ‘*anxious*' because ‘*the way we do things is different from what I've been doing in the Philippines*' but did not want to give colleagues the impression that ‘*I can't perform their job*'. Nonetheless, participants felt supported, and they could seek assistance, as illustrated by P14, ‘*You don't have the feeling of being afraid of asking since they will really help you*' and being ‘*encouraged to ask questions*' (P9), which was identified as facilitating to the adjustment.

Most participants felt well supported adjusting to their clinical areas as they had ‘*good staff development nurses*' (P2) and ‘*a lot of supportive staff around*' (P12), making it ‘*easy enough to ask questions*' (P2) as they ‘*are very happy to help you with any questions you have in mind*' (P8). Some participants who had experience working in metropolitan areas could not help but compare the differences in how well they were supported in a regional area compared to the city. Participants recalled experiencing ‘*bullying*' (P14), ‘*struggling because people around you are not approachable*' (P6) and feeling ‘*like a nobody*' (P3), which was opposite to their current experiences after relocating to a regional area.

Supernumerary shifts and orientation were deemed valuable as participants were ‘*not familiar with the paperwork*' (P13) or ‘*some protocols*' (P11). P10 described that they had ‘*a file for a new starter[s]*' that was a ‘go-to' file for ease of reference. Although some participants worked in a clinical area different to their prior experience, they stated that they did not have difficulty adjusting because ‘*we get [a] good education and good exposure before being put in a difficult situation*' (P9). P5's manager ‘*allowed at least a 2-week supernumerary so I could adjust very well with the system*'. Because of the ‘*educational opportunities*', P16 felt ‘*totally confident*' working in a different clinical area.

Some participants working in the clinical area, consistent with their experience, described having little difficulty transitioning. P13 stated, ‘*I have the experience [and] that helped me a lot*' and ‘*a lot of the floor nursing is the same*' (P17). Some participants ‘*felt it [was] a bit easier here*' (P11) because the ‘*systems are very good, very organised, [and] you can just pick any person to help you*' (P10). Most participants could also transfer their ‘*nursing skills easily enough*' (P17), although they needed to ‘*do the competency*' (P11) for other clinical skills. P7 stated, ‘*I just made sure that someone's looking after me, and when I'm confident, they're confident*' when describing the ease of skill transfer. Nonetheless, P12 welcomed ‘*go[ing] through competencies*', adding ‘*it was the best thing, because the machine, everything was different*'.

##### 4.2.2.2. Finding Meaningful Work Through Nurse Empowerment

All participants reported that their workplace encourages them to utilise, practice and develop their clinical skills. They felt satisfied that they could ‘*use [the] skills learned in [their] nursing studies*' (P9) and could do things that ‘*empower [their] autonomy as nurse[s]*' (P7). P6 added, ‘*If [I'm] not being supported [in my] skills, that would be a good factor that might put me to leave an organisation*'. They appreciated being ‘*selected and asked*' (P2) to do other roles and ‘*feel valued because [they are] learning more*' (P17). P16 recalled being ‘*allocated new staff [to] teach because they know I am teaching in university and they know my capacity and skill*'. P12 added, ‘*what[ever] capability you have, they'll take you and bring [it up] from you*'. The ‘*learning opportunities*' (P8) and chances to do ‘*different roles [one] can handle*' (P12) reinforced participants' aspirations to develop their clinical skills further and ‘*have more specialties [apart from] being a bedside nurse*' (P9). P16 reported having ‘*the same educational opportunity*' as everyone and being encouraged to participate in these. Most participants described that upskilling and ‘*learn[ing] something new is what motivates* [them] *to go to work*' (P7) and ‘*what* [they] *look forward to daily prior to going to a shift*' (P8) instead of ‘*do[ing] the same stuff every day*' (P12).

Most participants felt empowered when heard and counted as part of the team. They were encouraged to speak if they ‘*think something is wrong, even [to] the doctors*' (P9), as they ‘*always have the support of the doctor*' (P7) and ‘*most doctors are very easy to talk to*' (P2). Participants also described being able to do their jobs due to access to support and resources. P15 described being able to ‘*focus on patient care because everything [is] provided for*' compared to previous experiences. Having systems in place to manage ‘*risks at work related to [the] patient*' (P6), such as ‘*duress alarms and monitoring*' (P10), helped participants focus on doing their jobs. P6 added, ‘*The[y] provide us [with] everything that we need for work*', including ‘*time for education*' (P14), ‘*training*' (P11) for new equipment, and ‘*short-term courses*' (P10). The work environment in which participants were supported and had access to what they needed ‘*is a better place to work*' (P13), enabling them to ‘*make a difference in patient's lives*' (P15), ‘*help people*' (P1) and gain ‘*job satisfaction*' (P4). P16 recalled ‘*never go[ing] for an extra [shift]*' previously, but now at the current workplace, ‘*I'll go happily to help them whenever [they're] calling*'.

Additionally, most participants also reported being able to work ‘*part-time*' (P8) and do ‘*12-hour shifts*' (P9), which in a way, was described to give them a sense of control over their work patterns. P9 stated that 12-hour shifts are ‘*easier*' because ‘*if I do a short shift, [I] have to come [to work] every single day*'. Working part-time and doing 12-hour shifts allowed them to ‘*just work three days [instead] of five days in a week*' (P10), giving them ‘*rest time with family*' (P9) and ‘*enough [days] off*' (P16). P10 also opted to do a ‘*permanent night shift because I have a family to support during the day*', adding, ‘*my manager was very supportive, [providing] a mentor to work in some night shifts [with me]*'.

##### 4.2.2.3. Valuing Relationships at Work

All participants described the importance and effect of positive workplace relationships on their experiences. As participants spend a ‘*big percentage in [their] lives being at work*' (P15), they inevitably ‘*develop relationships*' (P17) with their colleagues, some of which are described as ‘*a really big factor in regards to staying in a workplace or not*' (P8). P5 recalled having ‘*offers for other nursing jobs*' but ‘*what keeps me on my current job is the people around me*'. The ‘*positive environment [which is] collaborative*' (P1) and ‘*supportive*' (P15) is ‘*very healthy and very good*' (P12) and ‘*made me want to continue to stay here because I could choose to do something different*' (P1). P5 added, ‘*It's very easy when you jive along with your co-workers because it's hard to work in an environment that's not supportive*'. ‘*Good relationships*' (P6) with colleagues could impact ‘*mental health*' (P10), and ‘*if colleagues are not assisting and [are] always complaining, it can cause stress and mental issues*' (P4). However, ‘*if [we] work together, [we] are happy, don't feel distressed*' (P6) and are not ‘*worried to come to work because [we] are happy to work*' (P10). P3 explained that ‘*if I see that I'm working with a certain group of people, I say I'm going to enjoy that day, but when I see [some others], I might just call in sick because [they're] lazy and [will] make me do all the things*'.

Most participants frequently mentioned the value of teamwork, with P2 stating, ‘*That's a big motivation when you've got a really good team around you*'. Working as ‘*part of the team*' (P7), ‘*if someone is busy, we are all helping them*' (P13). P4 added, ‘*If we don't get teamwork, it's like we compromise patient care*'. Being in a ‘*good team*' (P17), ‘*they always make sure that I'm getting a break*' (P11), ‘*ask every 10 minutes [if I] need help [when] I'm inside the isolated area*' (P16), and ‘*we [will] always be doing the shift well*' (P4). Having a ‘*supportive team [who] are always helping with everything*' (P11) ‘*makes work easier and makes you stay around for a longer time*' (P10). P14 described, ‘*When we work in that unit, we do really have teamwork; we just help each other, which I love*', adding, ‘*It's really a nice thing, and I would stay in this unit and hospital*'. Along with teamwork, participants valued ‘*respect*' (P7) and ‘*cooperati[on]*' (P4) amongst colleagues. P16 elaborated, ‘*They're not much knowledgeable about the neonate, so they will ask me to do it. If I have a doubt regarding the maternal side, I will call [them]*'.

Some participants also described their relationships with their patients and managers. Some participants ‘*treat patients as family*' (P6), contributing to ‘*job satisfaction because the patients are very happy to see us*' (P13). P9 stated, ‘*we get a bit low and don't get much motivation when patients are grumpy and not cooperating*', whereas ‘*it is motivat[ing] if patients are grateful and appreciative of what you have done*' (P14). In the same line as family treatment, some participants viewed their relationships with colleagues ‘*like a family*' (P5), with P3 adding, ‘*I have a work mum who is always encouraging'.* P6 added, ‘*Working in a unit is like a community, so it's good to build close relations with colleagues*'. Participants also described ‘*the unit manager [as] the best person*' (P13), as they can be ‘*approach[ed] at any time*' (P12) and are ‘*keeping things transparent*' (P2). Aside from being ‘*approachable*' (P16), the ‘*manager doesn't allow us to be mentally and physically fatigued, giv[ing] us mental and stress leave if we want*' (P3). ‘*Giv[ing] a lot of considerations to [the] nurses*' (P8), participants stated their managers were ‘*always try[ing] to do things that help us staff members*' (P9), ‘*listen to us when we need something*' (P14) and are ‘*always try[ing] to get new things [for us to] give us more confidence*' (P13).

#### 4.2.3. Theme 3: Integrating With the Community

##### 4.2.3.1. Embracing the Regional Lifestyle

Participants decided to settle not only in their place of employment but also in the host country and the regional area. Location and lifestyle were identified as crucial factors in participants choosing to stay in a regional area. Described as a ‘*great spot [to] land in*' (P2) and a ‘*middle ground*' (P15), most participants enjoy the ‘*laidback life*' (P6) and the ability to ‘*do nature tripping and be close to the beach*' (P5) but still be ‘*somewhere near the city*' (P7). They enjoy the ‘*places to visit in terms of beaches and the forest*' (P8), with P6 adding, ‘*you can go everywhere with ease*'. The regional area ‘*really suits our lifestyle*' (P5), where ‘*everything is nearby*' (P13). Previously having to ‘*always deal with traffic*' (P5), most participants now ‘*can't tolerate 1 hour travelling*' (P10) and ‘*don't want to spend 2 hours on the road to go and come from work*' (P12). The convenience of ‘*driving kids to school within 5 minutes*' (P10), ‘*[having work] just 5 minutes away from home*' (P14), and ‘*get[ting] everything within 10 minutes*' (P13) frees up a lot of time in the participants' days. This affords them ‘*a lot more free time*' (P17) ‘*to do all family things and spend much more time with the kids*' (P16) despite working full-time, with participants describing their lifestyle as ‘*a balance [of] work and life*' (P3).

All participants enjoy the ‘*slower pace of life*' (P1) compared to the city where ‘*It's busy and time is running*' (P16). While others describe the region as a ‘*sweet spot*' (P7) where ‘*we already have what we need*' (P10), some participants stated the downsides include ‘*not [having] a lot of shopping centres and food stores*' (P8) and ‘*having to go to the city for [some] health and medical treatments*' (P1). Nonetheless, other participants stated, ‘*[it's] only a short drive to the city*' (P6) and that they can ‘*just visit [rather than] move*' (P10). Other participants, however, considered the possibility of moving to the city for ‘*exposure to good schools*' (P9) and ‘*[opportunities] to work [in a] tertiary hospital*' (P8).

Aside from ease of travel and work–life balance, the weather and community relations were also mentioned as contributors to a good lifestyle. P4 stated, ‘*[my husband] couldn't adjust [to] the weather [before] and was in depression*'. P12 added ‘*the weather was affecting life*' because previously, ‘*we can't even go outside because of the bad weather*'. Participants are ‘*enjoying a bit more freedom*' (P11) to ‘*express [them]selves*' (P3) and ‘*[live] the life [they] would like to have*' (P14). P4 enjoys their current ‘*standard of living*' in which ‘*life has changed a lot from when we were in a Gulf country to over here*', adding ‘*that was the best decision in my life*'.

All participants have also integrated into their community, participating in ‘*church events*' (P15), ‘*cultural activities*' (P16) and ‘*sports*' (P11). Regional communities are described as ‘*more friendly and much more open to helping you with anything*' (P1) compared to the city where it is ‘*congested*' (P12), ‘*chaotic*' (P10) and ‘*some [people] don't really help*' (P14). Additionally, the ability to uphold their culture through socialising with people from the same source country lets them not ‘*completely leave [their] culture behind*' (P9), enabling them to ‘*mingle with [the] culture here as well*' (P9). It also enables their children who migrated young to still ‘*be part of [their] culture*' (P17). P13 described enjoying a ‘*community programme celebrating events [at least] five times a year with families from [India]*'. All participants have built their group of friends, now described as ‘*like a family*' (P5), that they can rely on for anything. P14 stated, ‘*You feel like you are still in [the] Philippines, like you still have your brothers and sisters*'. Regarding the overall community, P15 described ‘*not feel[ing] alienated because there's so much migrants around and Australia is very welcoming to migrants*'. P3 stated that being a ‘*multi-cultural country, you're not ashamed to do your own [things] like what you're used to when you're in the Philippines'*, adding, ‘*it's easier to live here, really*'. Participants' families are also ‘*loving it*' (P1), are ‘*happy to live in [the] region*' (P13) and have been ‘*putting [their] roots down*' (P17).

Being in the regional area where ‘*houses are 40% cheaper*' (P1), most participants were able to buy a house within three years or so after relocation. P13 described, ‘*I bought a house last year*' despite only ‘*living in [the area for the] last [few] years*'. The affordability and size of houses let ‘*you live like an Indian standard with space for garden and everything*' (P12). Participants were ‘*still able to save despite all [other] expenses*' (P14), attributing ‘*the cost of living in [the regional area] is not as high compared to [others]*' (P3). As participants are ‘*well compensated*' (P15) and ‘*paid [for] whatever we are doing [like] extra nights or extra hours*' (P16), they feel ‘*financially stable*' (P6) and are able to ‘*work [fewer] days rather than work full-time*' (P2). P11 stated that even ‘*if [my husband] has a small job, we can manage and we still feel comfortable*'. Most participants sum up embracing their regional lifestyle, saying, ‘*My life is here now. I enjoy my work. I love the places. I like the people around me. I have no plans to move out*' (P6) and ‘*This was all I was looking for when I was moving; I'm happy*' (P12). P3 shared a similar feeling, stating, ‘*This is my home. I don't treat my home [country] as my home anymore*'.

## 5. Discussion

This paper reports migrant nurses' perceptions of how their needs for autonomy, competence and relatedness were met throughout their migration and transition experiences. It also relates how migrant nurses perceived that these contributed to regional workplace retention.

### 5.1. Autonomy

Autonomy is the need for individuals to have a sense of ownership and self-endorse their actions [14]. Autonomy in the context of SDT is different from independence and freedom; instead, autonomy can be satisfied despite doing tasks that are required and not inherently enjoyable as long as the individuals understand the rationale and endorse the value of such tasks [14, 54]. Consistent with this, this study found that migrant nurses valued their work, took responsibility for their transition and understood the requirement to be assessed on certain clinical skills to ensure their competency was at par with the host country's standards. Thus, migrant nurses in this study accepted the need to undergo the required training and competencies without hesitation as they transitioned into their new roles and working environment.

This study also found that the ability to voice their clinical opinions and ask questions provided a sense of control over their everyday work, which may satisfy migrant nurses' need for autonomy. This is consistent with previous studies on nonmigrant nurses stating that a supportive work environment in which nurses can influence their workflow, make their own goals and contribute to the changes in their workplace culture was found to satisfy nurses' need for autonomy [29, 55]. This may also reflect professional autonomy as they exercise their clinical judgement and make decisions related to patient care within their professional boundaries [56, 57]. Additionally, the ability to choose their work patterns, such as working part-time or 12-hour instead of 8-hour shifts, meant that they may have more control over their time, balancing their time between work and home.

The study findings show that the new requirements of their role resulted in some migrant nurses challenging themselves to undertake roles that may be considered culturally inappropriate in their source countries, such as speaking up to authority (including doctors) and being proactive in their care delivery, such as initiating or suggesting a plan of care for their patients instead of passively waiting for doctors' orders. Other studies note conflict in professional and cultural expectations of migrant nurses, such as a female nurse questioning male doctors [58–60]. This conflict was not noted in this study as Australian hospitals are generally accepting of cultural diversity among staff [60]. Instead, migrant nurses seemed to accept their new roles and reconcile their professional and cultural habits, with many welcoming and adopting this change. This is consistent with findings reporting that migrant nurses develop strategies such as separate work personas, acquiring cultural behaviours and identifying practices they wish to adopt and the extent of this adoption [58, 61, 62]. The reconciliation of professional and cultural roles may impact integration in the host country, which may ultimately impact retention [63, 64], although this is not an issue identified in this study.

This study found that migrant nurses' need for autonomy may be satisfied when they can make decisions that align with their goals and circumstances despite experiencing various challenges. For instance, despite being able to migrate with family, some migrant nurses chose to migrate ahead of their families to enable them to take actions that otherwise would be too hard if they migrated altogether, such as renting rooms in shared houses or working longer hours. Additionally, although migrant nurses were employed in a regional area to meet visa requirements or as they were unable to obtain employment in metropolitan areas, the affordability, welcoming and ‘slow-paced' nature of the regional area afforded the migrant nurses in this study the opportunity to follow the lifestyle they prefer, which may have further satisfied their need for autonomy, and thereby contributed to their decision to stay.

### 5.2. Competence

Competence is the need to feel proficient and successful in one's endeavours [14]. Migration may challenge migrant nurses' perceptions of competence as they experience unfamiliar cultures, languages and professional environments. The differences in professional registration requirements across countries often result in migrant nurses undertaking regulatory exams, transition programs or further university training to gain registration [10, 65, 66]. This study found that unfamiliar clinical routines and norms and differences in communication styles were challenging for migrant nurses, especially those who did not undergo transition programs. These findings were evident in other studies where differences in communication styles, colloquialisms, informal language, conversational speeds and varied accents rather than the language itself posed a challenge, resulting in migrant nurses' doubts in their professional credibilities and frustrations as they attempt to communicate [4, 67].

This study demonstrated that flexible supernumerary days, supportive work environments where questions were welcomed, and effective mentorship facilitated migrant nurses' adjustment and enabled them to transition effectively. Insufficient support is an identified barrier to migrant nurses' transition, with migrant nurses being reluctant to make clinical decisions [68, 69], although this was not identified in the current study. Instead, some participants reported that the regional area provided better organisational support and mentorship than they have received in metropolitan areas. While migrant nurses were cautious of their practice and concerned about their colleagues' perceptions of their clinical skills, they attributed their supportive workplace to facilitating the ease of transition and supporting their confidence, particularly in relation to decision-making and clinical competencies.

Moreover, professional development in the form of educational opportunities, training, short courses and opportunities to work in new clinical areas and, thus, new roles with support may also contribute to satisfying their need for competence. Differences in practice, such as decision-making, contributed to empowerment and positive feelings towards the workplace. Similar findings reported that migrant nurses are empowered by their professional development [70]. Other studies identified inequality in opportunities for career advancement and downward occupational mobility where migrant nurses could not practice in their area of expertise, resulting in deskilling and devaluing [4, 64]. However, the current study found no similar findings; migrant nurses were satisfied with their professional practice and growth, and these factors contributed to their desire to stay in their regional workplace.

This study found migrant nurses adapting to the new lifestyle, adjusting to the culture and overcoming challenges, which may support their need for competence by increasing perceptions of self-efficacy. Self-efficacy involves developing the required coping mechanisms, such as patience, resilience, determination and independence, as an individual adjusts to the community and workplace [71]. Moreover, being in a regional area enabled migrant nurses in this study to enjoy financial stability as the cost of living is less than in metropolitan areas. This further met their need for competence as they perceived themselves to be transitioning successfully and settling into life and work in a regional area.

### 5.3. Relatedness

Relatedness is the need to belong, have meaningful connections and feel valued and supported by others [14]. This study found that hospital orientation, organisational support and connections with peers from the same source country may promote a sense of belongingness among migrant nurses, especially during the early stages of transition. This supports evidence that organisational support through mentorship, orientation programs and collegial support assists migrant nurses in navigating the challenges during migration and transition [63, 72]. Positive working relationships and connections with peers enable migrant nurses to draw social support from their colleagues during workplace challenges, promoting resilience and assisting acculturation [4, 26].

This study showed that migrant nurses found it easier to connect with others they believed understood their unique challenges—fellow migrant nurses. Unfamiliarity with a new culture may cause feelings of being an outsider [58]; thus, connecting with nurses from similar backgrounds offers a sense of familiarity and kinship. Working alongside and being mentored by peers from similar backgrounds provides a sense of belonging, thereby mitigating isolation and anxiety and enabling migrant nurses to cope with adversities [61, 70].

This study demonstrated that the need for relatedness satisfied through workplace relationships and interactions, manager and peer support, and effective teamwork contributes to migrant nurses' psychological well-being and desire to stay in their workplace. Feedback and appreciation from patients, colleagues, and managers contribute to a sense of belonging [61]. This supports studies purporting that satisfactory working conditions and supportive work environments contribute to retention among migrant nurses [64, 73]. Nonetheless, those with relatedness needs unsatisfied at work may seek support systems outside of work to meet these needs [26]. This emphasises migrant nurses' needs for support networks within and outside the workplace.

This study found that most migrant nurses' support networks outside work comprised their immediate families and people from similar backgrounds. Family presence is well known to contribute to migrant nurse retention in the host country, with migrant nurses prioritising their families' overall adjustment over their professional aspirations [64, 74]. Workplace relationships may serve as a bridge, welcoming migrant nurses to community and cultural events where they could build more relationships with others. While other studies found that migrant nurses had difficulty building supportive relationships [67], this study identified that being in a regional area facilitated workplace and community relationships much easier. Migrant nurses in this study who previously worked and lived in metropolitan areas reported that people in regional areas are generally more welcoming and willing to help than in the city. The ‘more personal' and ‘calmer nature' of regional areas enabled them to integrate and build deeper connections in their communities. Rural communities were also reported to be welcoming and accepting of migrant nurses [68]. Feelings of belonging in the community and workplace may contribute to meeting migrant nurses' needs for relatedness and decisions to settle in their workplace and regional areas.

## 6. Strengths and Limitations

The single hospital setting presents a limitation, as experiences may differ in other settings (e.g., academia, aged care, clinic, community, private sectors or other hospitals). While this limits generalisability, it also presents opportunities for further studies involving other geographic locations, settings and other migrant professionals and workers or nonmigrant nurses. The sample may not have included participants whose organisational experiences were unfavourable as they may have been hesitant to speak about their workplace. The heterogeneity of the migrant nurse population presents another limitation, as cultural, personal, or professional differences (different areas of clinical practice) and previous history of migration may impact perceptions of their transition experiences. Additionally, this study did not explicitly discuss retention, although it related migrant nurses' perceptions of how their overall experiences contribute to retention. Nonetheless, the methods employed throughout the study were in line with Lincoln and Guba's [50] framework for rigour, ensuring credibility, dependability, confirmability and transferability.

## 7. Implications for Policy and Practice

This research reports migrant nurses' transition experiences through the SDT perspective, expanding on knowledge of migrant nurses' challenges and strategies that may mitigate these. While migrant nurses are responsible for adapting, organisations also play a role in successful integration [67, 69]. Organisational support during relocation, such as accommodation and transport and connection with peers from similar backgrounds, is vital to the transition of migrant nurses. Organisations employing migrant nurses should provide specific orientation programs tailored to meet migrant nurses' needs. These programs may include organisational standards, policies, common terminologies, documentation and social, language and cultural training [63, 68, 74], which are in addition to the standard orientation programs they provide to all employees. Structured orientation programs, peer mentoring and other clinical training are essential, shaping their initial experiences in the organisation, which is found to contribute to organisational commitment and decreased turnover intention [10, 69, 74]. Connecting with peers from similar backgrounds may serve as a valuable support, mitigating challenges and facilitating workplace and community transition. Additionally, supportive work environments alleviate migrant nurses' doubts in their abilities, demonstrating their competence as they take control of their new roles and develop self-efficacy.

Healthcare organisations in regional areas can capitalise on the lifestyle their regions offer, enabling their nurses to have work arrangements conducive to work–life balance. As migrant nurses concurrently adapt to the workplace and the community, the ability to spend quality time outside of work with their families and support networks may improve migrant nurses' perceptions of their overall lifestyle, which may subsequently impact their intention to stay in the workplace and the area.

## 8. Conclusion

Migrant nurses experience unique challenges that may hinder successful transition and integration in the workplace and community. The SDT perspective may offer valuable insights into migrant nurses' transition experiences that may impact their long-term retention. Orientation programs tailored to cater to migrant nurses' needs, such as nuances in communication and differences in role expectations, may promote workplace transition. A supportive workplace, flexible work arrangements, professional development, positive workplace interactions and support from peers with similar backgrounds may satisfy migrant nurses' needs for autonomy, competence and relatedness, which, in turn, may promote intention to stay and contribute to a more stable regional workforce. While there are limitations to living in regional areas, they may offer a calm and welcoming atmosphere appealing to migrant nurses, facilitating successful workplace transition and community integration, which may influence their intention to stay in the area.

## Data Availability

Data are available on request due to privacy/ethical restrictions.

## Supporting Information

Appendix S1 (Supporting information). Interview guide for this study.

## References

[1] O’Brien B. C., Harris I. B., Beckman T. J., Reed D. A., Cook D. A. (2014). Standards for Reporting Qualitative Research: A Synthesis of Recommendations. *Academic Medicine*.

[2] International Council of Nurses (2021). ICN Policy Brief–The Global Nursing Shortage and Nurse Retention. https://www.icn.ch/sites/default/files/inline-files/ICNPolicyBrief_NurseShortageandRetention_0.pdf.

[3] Buchan J., Catton H., Shaffer F. A. (2022). Sustain and Retain in 2022 and Beyond: The Global Nursing Workforce and the COVID-19 Pandemic. https://www.intlnursemigration.org/wp-content/uploads/2022/01/Sustain-and-Retain-in-2022-and-Beyond-The-global-nursing-workforce-and-the-COVID-19-pandemic.pdf.

[4] Pressley C., Newton D., Garside J., Simkhada P., Simkhada B. (2022). Global Migration and Factors that Support Acculturation and Retention of International Nurses: A Systematic Review. *International Journal of Nursing Studies Advances*.

[5] Jones A., Rahman R. J., Jiaqing O. (2019). A Crisis in the Countryside–Barriers to Nurse Recruitment and Retention in Rural Areas of High-Income Countries: A Qualitative Meta-Analysis. *Journal of Rural Studies*.

[6] McCallum A. M., Vandenberg H. E. R., Penz K. L. (2023). Help Wanted, Experience Preferred, Stamina a Must: A Narrative Review of the Contextual Factors Influencing Nursing Recruitment and Retention in Rural and Remote Western Canada From the Early Twentieth Century to 2023. *Canadian Journal of Nursing Research*.

[7] Rajbangshi P. R., Nambiar D., Choudhury N., Rao K. D. (2017). Rural Recruitment and Retention of Health Workers Across Cadres and Types of Contract in North-East India: A Qualitative Study. *WHO South-East Asia Journal of Public Health*.

[8] Government of Canada (2024). Immigrate as a Provincial Nominee. https://www.canada.ca/en/immigration-refugees-citizenship/services/immigrate-canada/provincial-nominees.html.

[9] Kandel W. A., Wilson J. H., Heisler E. J. (2023). *Immigration Options and Professional Requirements for Foreign Health Care Workers*.

[10] Villamin P., Lopez V., Thapa D. K., Cleary M. (2024). Retention and Turnover Among Migrant Nurses: A Scoping Review. *International Nursing Review*.

[11] Organisation for Economic Co-Operation and Development (2023). Health Workforce Migration: Foreign-Trained Nurses by Country of Origin–Stock. https://stats.oecd.org.

[12] Villamin P., Lopez V., Thapa D. K., Cleary M. (2023). Nurse Migration to Australia: Past, Present, and Future. *Collegian*.

[13] Ryan R. M., Deci E. L. (2000). Self-Determination Theory and the Facilitation of Intrinsic Motivation, Social Development, and Well-Being. *American Psychologist*.

[14] Rigby C. S., Ryan R. M. (2018). Self-Determination Theory in Human Resource Development: New Directions and Practical Considerations. *Advances in Developing Human Resources*.

[15] Ryan R. M., Deci E. L. (2020). Intrinsic and Extrinsic Motivation From a Self-Determination Theory Perspective: Definitions, Theory, Practices, and Future Directions. *Contemporary Educational Psychology*.

[16] Van den Broeck A., Ferris D. L., Chang C.-H., Rosen C. C. (2016). A Review of Self-Determination Theory’s Basic Psychological Needs at Work. *Journal of Management*.

[17] Martela F., Ryan R. M. (2023). Clarifying Eudaimonia and Psychological Functioning to Complement Evaluative and Experiential Well-Being: Why Basic Psychological Needs Should Be Measured in National Accounts of Well-Being. *Perspectives on Psychological Science*.

[18] Vansteenkiste M., Ryan R. M. (2013). On Psychological Growth and Vulnerability: Basic Psychological Need Satisfaction and Need Frustration as a Unifying Principle. *Journal of Psychotherapy Integration*.

[19] Onyishi I. E., Enwereuzor I. K., Ogbonna M. N., Ugwu F. O., Amazue L. O. (2019). Role of Career Satisfaction in Basic Psychological Needs Satisfaction and Career Commitment of Nurses in Nigeria: A Self-Determination Theory Perspective. *Journal of Nursing Scholarship*.

[20] Ni Y.-X., Wu D., Bao Y., Li J.-P., You G.-Y. (2023). The Mediating Role of Psychological Needs on the Relationship Between Perceived Organizational Support and Work Engagement. *International Nursing Review*.

[21] Trépanier S.-G., Fernet C., Austin S., Forest J., Vallerand R. J. (2014). Linking Job Demands and Resources to Burnout and Work Engagement: Does Passion Underlie These Differential Relationships?. *Motivation and Emotion*.

[22] Klein D. S. (2017). *The Effect of Hospital Nurse Basic Psychological Needs Satisfaction on Turnover Intention and Compassion Fatigue*.

[23] Bai C., Bai B., Kong F. (2021). Strength Use and Nurses’ Depressive Symptoms: The Mediating Role of Basic Psychological Needs Satisfaction. *Journal of Nursing Management*.

[24] Gillet N., Fouquereau E., Coillot H. (2018). The Effects of Work Factors on Nurses’ Job Satisfaction, Quality of Care and Turnover Intentions in Oncology. *Journal of Advanced Nursing*.

[25] Deci E. L., Ryan R. M. (2000). The “What” and “Why” of Goal Pursuits: Human Needs and the Self-Determination of Behavior. *Psychological Inquiry*.

[26] Magson N. R., Craven R. G., Ryan R. M. (2022). A Cross-Cultural Investigation of Basic Psychological Need Satisfaction at Work in an Indigenous and Non-Indigenous Australian Sample Across Occupation Types. *Journal of Cross-Cultural Psychology*.

[27] Ahlstedt C., Lindvall C. E., Holmström I. K., Muntlin Å. (2020). Flourishing at Work: Nurses’ Motivation Through Daily Communication–An Ethnographic Approach. *Nursing and Health Sciences*.

[28] Burstyn I., Holt K. (2022). Pride and Adversity Among Nurses and Physicians During the Pandemic in Two US Healthcare Systems: A Mixed Methods Analysis. *BMC Nursing*.

[29] Koivisto J.-M., Multisilta J., Haavisto E. (2021). Surgical Nurses’ Experiences With Intrinsic Work Motivation: A Focus on Autonomy, Competence and Relatedness. *Hoitotiede*.

[30] Li H., Nie W., Li J. (2014). The Benefits and Caveats of International Nurse Migration. *International Journal of Nursing Science*.

[31] Kurup C., Burston A., Betihavas V., Jacob E. R. (2024). Internationally Qualified Nurses’ Perspectives on Transitioning Specialty Skills Within Australia: A Content Analysis. *Nursing Open*.

[32] Randall P. S., De Gagne J. C. (2023). Supporting Self-Determination Among Internationally Educated Nurses: A Discussion. *Contemporary Nurse*.

[33] Villamin P., Lopez V., Thapa D. K., Cleary M. (2025). Why Did They Migrate Here’?: A Qualitative Descriptive Study Exploring Nurses’ Motivations for Migration and Regional Relocation. *Journal of Advanced Nursing*.

[34] Sandelowski M. (2010). What’s in a Name?. *Research in Nursing & Health*.

[35] Guba E. G., Lincoln Y. S., Denzin N. K., Lincoln Y. S. (1994). Competing Paradigms in Qualitative Research. *Handbook of Qualitative Research*.

[36] Kivunja C., Kuyini A. B. (2017). Understanding and Applying Research Paradigms in Educational Contexts. *International Journal of Higher Education*.

[37] Bradshaw C., Atkinson S., Doody O. (2017). Employing a Qualitative Description Approach in Health Care Research. *Global Qualitative Nursing Research*.

[38] Roudsari R. L. (2019). Qualitative Description: A Less Sophisticated Approach for Junior Qualitative Researchers. *Journal of Midwifery & Reproductive Health*.

[39] Villamin P., Lopez V., Thapa D. K., Cleary M. (2024). A Worked Example of Qualitative Descriptive Design: A Step-by-step Guide for Novice and Early Career Researchers. *Journal of Advanced Nursing*.

[40] Coleman J. S. (1958). Relational Analysis: The Study of Social Organizations With Survey Methods. *Human Organization*.

[41] Raifman S., DeVost M. A., Digitale J. C., Chen Y.-H., Morris M. D. (2022). Respondent-Driven Sampling: A Sampling Method for Hard-To-Reach Populations and Beyond. *Current Epidemiology Reports*.

[42] Cleary M., Hungerford C., Johnston-Devin C., West S., Jackson D. (2024). Keeping the ‘Quality’ in Qualitative Research: Embracing Technology to Enhance Participation in Qualitative Interviews. *Journal of Advanced Nursing*.

[43] Morse J. M. (1995). The Significance of Saturation. *Qualitative Health Research*.

[44] Braun V., Clarke V. (2006). Using Thematic Analysis in Psychology. *Qualitative Research in Psychology*.

[45] Braun V., Clarke V. (2022). *Thematic Analysis: A Practical Guide*.

[46] Doyle L., McCabe C., Keogh B., Brady A., McCann M. (2020). An Overview of the Qualitative Descriptive Design Within Nursing Research. *Journal of Research in Nursing*.

[47] Vaismoradi M., Turunen H., Bondas T. (2013). Content Analysis and Thematic Analysis: Implications for Conducting a Qualitative Descriptive Study. *Nursing and Health Sciences*.

[48] Braun V., Clarke V. (2020). Can I Use TA? Should I Use TA? Should I Not Use TA? Comparing Reflexive Thematic Analysis and Other Pattern-Based Qualitative Analytic Approaches. *Counselling and Psychotherapy Research*.

[49] Sandelowski M. (1995). Qualitative Analysis: What it is and How to Begin. *Research in Nursing & Health*.

[50] Lumivero (2023). NVivo 14 (For Mac). *Computer Software*.

[51] Lincoln Y. S., Guba E. G. (1985). *Naturalistic Inquiry*.

[52] Milne J., Oberle K. (2005). Enhancing Rigor in Qualitative Description: A Case Study. *The Journal of Wound, Ostomy and Continence Nursing*.

[53] Morse J. M. (2015). Critical Analysis of Strategies for Determining Rigor in Qualitative Inquiry. *Qualitative Health Research*.

[54] Deci E. L., Olafsen A. H., Ryan R. M. (2017). Self-Determination Theory in Work Organizations: The State of a Science. *Annual Review of Organizational Psychology and Organizational Behavior*.

[55] Ahlstedt C., Lindvall C. E., Holmström I. K., Athlin Å. M. (2019). What Makes Registered Nurses Remain in Work? An Ethnographic Study. *International Journal of Nursing Studies*.

[56] Liebenberg J.-M., Scholtz S. E., De Beer L. T. (2022). The Daily Basic Psychological Need Satisfaction and Work Engagement of Nurses: A ‘Shortitudinal’ Diary Study. *Healthcare*.

[57] Elahi N., Rouhi-Balasi L., Ebadi A., Jahani S., Hazrati M. (2020). Professional Autonomy of Nurses: A Qualitative Meta-Synthesis Study. *Iranian Journal of Nursing and Midwifery Research*.

[58] Balante J., Broek D. v. d., White K. (2021). How Does Culture Influence Work Experience in a Foreign Country? An Umbrella Review of the Cultural Challenges Faced by Internationally Educated Nurses. *International Journal of Nursing Studies*.

[59] Konno R. (2006). Support for Overseas Qualified Nurses in Adjusting to Australian Nursing Practice: A Systematic Review. *International Journal of Evidence-Based Healthcare*.

[60] O’Callaghan C., Loukas P., Brady M., Perry A. (2019). Exploring the Experiences of Internationally and Locally Qualified Nurses Working in a Culturally Diverse Environment. *Australian Journal of Advanced Nursing*.

[61] Safari K., McKenna L., Davis J. (2022). Transition Experiences of Internationally Qualified Health Care Professionals: A Narrative Scoping Review. *International Journal of Nursing Studies*.

[62] Skaria R., Whitehead D., Leach L., Walshaw M. (2019). Experiences of Overseas Nurse Educators Teaching in New Zealand. *Nurse Education Today*.

[63] Rajpoot A., Merriman C., Rafferty A.-M., Henshall C. (2024). Transitioning Experiences of Internationally Educated Nurses in Host Countries: A Narrative Systematic Review. *International Journal of Nursing Studies Advances*.

[64] Ung D. S. K., Goh Y. S., Poon R. Y. S. (2024). Global Migration and Factors Influencing Retention of Asian Internationally Educated Nurses: A Systematic Review. *Human Resources for Health*.

[65] Crea-Arsenio M., Baumann A., Blythe J. (2023). The Changing Profile of the Internationally Educated Nurse Workforce: Post-Pandemic Implications for Health Human Resource Planning. *Healthcare Management Forum*.

[66] John McKitterick D., Peters M. D. J., Corsini N., Chiarella M., Eckert M. (2021). International Nursing Students’ and International Nursing Graduates’ Experiences of Transition to the Nursing Workforce: A Systematic Review of Qualitative Evidence. *Nurse Education in Practice*.

[67] Bond S., Merriman C., Walthall H. (2020). The Experiences of International Nurses and Midwives Transitioning to Work in the UK: A Qualitative Synthesis of the Literature From 2010 to 2019. *International Journal of Nursing Studies*.

[68] Chun Tie Y., Birks M., Mills J. (2018). The Experiences of Internationally Qualified Registered Nurses Working in the Australian Healthcare System: An Integrative Literature Review. *Journal of Transcultural Nursing*.

[69] Kurup C., Burston A., Miles S. (2023). Transition of Internationally Qualified Nurses in Australia: Meta-Synthesis of Qualitative Studies. *Collegian*.

[70] Pung L.-X., Goh Y.-S. (2017). Challenges Faced by International Nurses When Migrating: An Integrative Literature Review. *International Nursing Review*.

[71] Ghazal L. V., Ma C., Djukic M., Squires A. (2020). Transition-to-U.S. Practice Experiences of Internationally Educated Nurses: An Integrative Review. *Western Journal of Nursing Research*.

[72] Nourpanah S. (2019). Maybe We Shouldn’t Laugh So Loud: The Hostility and Welcome Experienced by Foreign Nurses on Temporary Work Permits in Nova Scotia, Canada. *Labour*.

[73] Leone C., Dussault G., Rafferty A. M., Anderson J. E. (2020). Experience of Mobile Nursing Workforce From Portugal to the NHS in UK: Influence of Institutions and Actors at the System, Organization and Individual Levels. *The European Journal of Public Health*.

[74] Moyce S., Lash R., de Leon Siantz M. L. (2016). Migration Experiences of Foreign Educated Nurses: A Systematic Review of the Literature. *Journal of Transcultural Nursing*.

