# Supplementary material for: From Vulnerability to Stability: Migrant Nurses' Experiences of Autonomy, Competence and Relatedness—A Qualitative Descriptive Study
Source: J Nurs Manag. 2025 Mar 2;2025:8260066. doi: 10.1155/jonm/8260066 (PMC11985247; doi:10.1155/jonm/8260066)
Supplement: Supporting Information — Additional supporting information can be found online in the Supporting Information section. [file 8260066.f1.docx]

**Appendix S1 (Supplementary material).** Interview guide for this study.

Interview guide:

1. Demographics
2. How old are you?
3. What is your gender?
4. What is your marital status and current living arrangement?
5. Do you have any children?
   1. (For participants with children) How many children do you have? How old are they? Where are your children currently?
6. What year did you first obtain your nursing degree, and which country did you receive this from?
7. What is your highest nursing qualification? Do you have any diplomas, certificates, or have you completed post-graduate nursing or non-nursing related studies since moving to Australia (or do you plan on enrolling in any)?
8. Have you worked as a nurse in other countries (excluding Australia)? If so, which country/countries and for how long? Why did you leave?
9. What year did you migrate to Australia? What country did you migrate from?
10. Did you work in other states/regions in Australia before joining xxx? (If so, where and how long?) What year did you start working as a nurse at xxx?
11. Moving to Australia
12. Why did you migrate to Australia?

(Prompts)

- *What events or experiences made you decide to move?*
- *What motivated you to migrate?*
- *Who inspired you to migrate to Australia?*

1. What steps did you take to migrate to Australia as a nurse?

(Prompts)

- *What steps did you take to gain nursing registration?*
- *What steps did you take to obtain a working visa?*

1. What challenges did you anticipate before moving to Australia, and how did you prepare for these?
2. What challenges did you face, and how did you overcome them?
3. What strategies or support outside of employment helped you overcome these challenges?
4. Employment in Australia
5. Which clinical area are you currently working in? And is this in alignment with your qualifications, previous clinical area, or experience?

(Prompts)

- *How do you feel about working in a similar (or different) clinical area to your previous clinical area or experience?*

1. Do you feel that your current remuneration is adequate? Why or why not?
2. What support did you receive to help you adjust in your workplace?
   1. How do you feel about the organisational support you received?
   2. How prepared do you feel to do your job?
3. What has been your experience working as a nurse in Australia?

(Prompts)

- *How do your current and past experiences compare?*
- *How did you adjust to working as a nurse in Australia?*
- *Describe your job and day-to-day work life.*
- *How does being a nurse make you feel?*
- *How important is being a nurse to you?*
- *If you had another option, would you still continue to work as a nurse? Why or why not?*

1. Describe your thoughts and feelings surrounding your desire to continue to stay in your workplace.

(Prompts)

- *What factors motivate you at work?*
- *What rewards would motivate you to stay longer in your workplace?*
- *What is influencing you to stay?*
- *What job-related factors, events, or situations may prompt you to consider leaving?*

1. Describe how your workplace relationships and interactions affect you.

(Prompts)

- *How do you describe your relationship with your co-workers, patients, and manager?*
- *Do you feel understood or cared for by your peers?*
- *In what way do your workplace relationships and interactions affect your ability to do your job? Could you please provide a specific example or scenario?*
- *In what way do your workplace relationships and interactions affect your desire to stay in your workplace? Could you please provide a specific example or scenario?*

1. Who do you consider your greatest resource (or support) when you are dealing with challenges at work?
2. Overall life in Australia
3. In general, how would you describe your experience as a migrant in Australia? And how does this experience make you feel?

(Prompts)

- *What was your experience during your first month of migration to Australia?*
- *Did you have any support or assistance (employer, family, friends) to help you adjust to life in Australia?*
- *How different is your current experience and lifestyle now to your previous place of residence?*
- *Can you say that you have adapted to living in Australia? Why or why not?*

1. Looking back, you mentioned XXXXX motivated you to migrate. Do you still feel motivated by XXXXX?

(Prompts)

- *Does XXXXX still motivate you to stay?*

1. How would you describe your life in Australia, outside of your workplace?

(Prompts)

- *When not at work, how do you go about your day?*
- *Do you have family and friends nearby that you see often?*
- *How active are you in participating in social events?*
- *Are the people in your community generally friendly towards you?*

1. (For participants who migrated with their spouse) What is your spouse’s experience after moving to Australia?

(Prompts)

- *Is your spouse employed? If so, how does he/she feel about his/her current employment in Australia?*
- *Outside of work, is your spouse active in social, community, or sporting events?*

1. (For participants who migrated with their child/children) What is/are your child/children’s experience after moving to Australia?

(Prompts)

- *How is your child/children finding school here compared to where you were from?*
- *Is your child/children involved in community or school activities or sports?*

1. Closing Questions
   1. Is there anything related to the topic that you may not have thought about previously that occurred to you during this interview?
   2. Is there anything else you would like to say to help me understand how your experiences influence your intention to stay in your current workplace and place of residence?
